# Books Review

**Published:** 1871-02

**Authors:** 


### Books Review.

*The American Journal of Obstetrics and Diseases of Women and Children.*

We have received, through the liberality of the editor, beautifuly bound volumes of the American Journal of Obstetrics and Diseases of Women and Children, for which we desire to return, (since we can do nothing more,) our most hearty thanks. Vols. I and II, constitute, of themselves alone, an almost complete record of our present knowledge in this department, do fully represent the progress which has been made since the commencement of publication. The Journal is edited with consummate skill and ability, and receives contributions from many of the most eminent writers and teachers in the profession. The advance of knowledge in this department is so great that practitioners of medicine must give attention to present teaching, or soon find that their profession is far in advance of them, rather than themselves in advance of the profession. In no department of medicine can men, at the present time stand still, they must move forward in search of greater knowledge or slide backward into deeper ignorance. This Journal is a necessity in its department, since in it have appeared, and constantly are appearing, important practical papers, singly of great value, and combined, constituting the progress the art is making from month to month.

*Satan in Society.* By a Physician. Cincinnati and New York: C. F. Vent. Chicago: J. S. Goodman & Co. 1871.

Satan in Society is a book to be read and not to be reviewed very much. By this we do not mean that it will not bear reviewing, but that reading accounts of what it is, will in no way answer the craving of the popular soul to read it. It must be read by nearly all, since everybody is interested in the subjects it discusses and in the manner of treatment. Satan is in society in forms and disguises known only to a physician, and on this account the book must be read. Satan, alas! constitutes a part of so-called "Good Society," and a greater part of it than any one but a physician mistrusts, and on this account this book will bear reading. Satan may be said to have written himself up very well, and though his editor has not always treated him with the respect due his position in society, still, probably he can obtain his satanic pardon by penance and reform. Possibly some reader may yet ask what is it? It is Satan in Society, by a Physician, who mixes him up shamefully with Masturbation, Rights of Offspring, Physiology of Marriage, Woman without Christianity, Physiological Comparison of the Sexes. What can woman do in the world, Prostitution, Happiness in Wedlock, Conjugal Aphorisms, and ten thousand other subjects into which we should suppose Satan would refuse to be associated, but as he has allowed himself to be "drawn in," he must now suffer the disgrace of it.

But again, and last of all, we assure our readers that it is a book to be read, it must be read, the "Satan in Society" can't prevent its being read.

---

*First Medical and Surgical Report of the Boston City Hospital.* Edited by J. NELSON BORLAND, Physician; DAVID W. CHEEVER, Surgeon. Boston: LITTLE, BROWN & CO.

Tnis is a magnificent volume of near seven hundred pages, containing a very instructive and well written medical and surgical history of the Institution. It comprises cases illustrative of almost every form of disease and nearly every condition requiring surgical interference. The cases requiring it are very beautifully illustrated with wood cuts and chromo-lithographic plates. The work reflects the highest credit upon the Editors and upon the Institution, and is of great value to the profession.

